# Increase in anaphylaxis-related hospitalizations but no increase in fatalities: An analysis of United Kingdom national anaphylaxis data, 1992-2012

**DOI:** 10.1016/j.jaci.2014.10.021

**Published:** 2015-04

**Authors:** Paul J. Turner, M. Hazel Gowland, Vibha Sharma, Despo Ierodiakonou, Nigel Harper, Tomaz Garcez, Richard Pumphrey, Robert J. Boyle

**Affiliations:** a Section of Paediatrics (Allergy & Immunology) and MRC & Asthma UK Centre in Allergic Mechanisms of Asthma, Imperial College London, London, United Kingdom; b Division of Paediatrics and Child Health, University of Sydney, Sydney, Australia; c Allergy Action & Anaphylaxis Campaign UK, Farnborough, United Kingdom; d Central Manchester University Hospitals NHS Foundation Trust, Manchester, United Kingdom

**Keywords:** Anaphylaxis, drug allergy, epidemiology, food allergy, hospitalization, insect sting allergy, ASR, Age-standardized rate, ICD, International Classification of Diseases, ONS, Office for National Statistics, UK, United Kingdom

## Abstract

**Background:**

The incidence of anaphylaxis might be increasing. Data for fatal anaphylaxis are limited because of the rarity of this outcome.

**Objective:**

We sought to document trends in anaphylaxis admissions and fatalities by age, sex, and cause in England and Wales over a 20-year period.

**Methods:**

We extracted data from national databases that record hospital admissions and fatalities caused by anaphylaxis in England and Wales (1992-2012) and crosschecked fatalities against a prospective fatal anaphylaxis registry. We examined time trends and age distribution for fatal anaphylaxis caused by food, drugs, and insect stings.

**Results:**

Hospital admissions from all-cause anaphylaxis increased by 615% over the time period studied, but annual fatality rates remained stable at 0.047 cases (95% CI, 0.042-0.052 cases) per 100,000 population. Admission and fatality rates for drug- and insect sting–induced anaphylaxis were highest in the group aged 60 years and older. In contrast, admissions because of food-triggered anaphylaxis were most common in young people, with a marked peak in the incidence of fatal food reactions during the second and third decades of life. These findings are not explained by age-related differences in rates of hospitalization.

**Conclusions:**

Hospitalizations for anaphylaxis increased between 1992 and 2012, but the incidence of fatal anaphylaxis did not. This might be due to increasing awareness of the diagnosis, shifting patterns of behavior in patients and health care providers, or both. The age distribution of fatal anaphylaxis varies significantly according to the nature of the eliciting agent, which suggests a specific vulnerability to severe outcomes from food-induced allergic reactions in the second and third decades.

Acute, life-threatening systemic allergic reactions (anaphylaxis) can lead to cardiorespiratory arrest within minutes.^1^ For those affected, the threat of further episodes can lead to significant lifestyle restrictions and psychological consequences.^2,3^ Recent Australian data suggest that episodes of anaphylaxis, particularly those triggered by drugs or food, might be increasing.^4^ Recent US data suggest that at least 1.6% of the population have a history of anaphylaxis,^5^ although this might be an overestimate.^6^ Although anaphylaxis is a relatively common occurrence, at least as defined by the patient, severe immediate outcomes, such as fatality or admission to an intensive care unit, are rare.^6-8^ Indeed, a recent report from a European anaphylaxis registry relating to severe anaphylaxis found that only 2% of more than 3000 cases of significant anaphylaxis involved cardiorespiratory arrest.^9^

For this reason, there is a paucity of data relating to trends in fatal (or near-fatal) allergic reactions over time. The apparent increase in hospitalization caused by anaphylaxis might be due to a real increase in disease or a change in health care provider or patient behavior, such as improved management. A parallel increase in fatal anaphylaxis incidence would provide supportive evidence for the former. Therefore we assessed trends in hospital admissions and fatalities caused by anaphylaxis in a population-based data set that includes the largest reported series of cases of fatal anaphylaxis. We explored time trends in all-cause and specific-cause anaphylaxis events in different age groups and by sex to understand which patients are at highest risk for severe allergic reactions. Previous small case series suggest that teenagers and young adults might be at the highest risk of fatal food-induced anaphylaxis for unknown reasons,^10^ but this has not been confirmed in large population-based data sets and might be confounded by age-related differences in the incidence of anaphylaxis.

## Methods

We examined time trends and age/sex distribution for hospital admissions and fatalities caused by anaphylaxis in England and Wales over a 21-year period (1992-2012). We further analyzed the data for differences in time trend and age distribution by triggering agent (food, iatrogenic causes [eg, oral and parenteral medication and contrast media], and insect stings).

### Anaphylaxis admission data

Hospital admission data for England and Wales are collated by the Hospital Episodes Statistics database (coordinated through the Health & Social Care Information Centre) and the Patient Episode Database for Wales (National Health Service Wales Informatics Service), respectively. We extracted data relating to hospitalizations in which anaphylaxis was the primary diagnosis for the calendar years 1992 to 2012. We did not include emergency department visit that did not result in a hospital admission. For analysis of trigger-specific age distribution, we limited data extraction to the years 1998 to 2012 to avoid confounding because of International Classification of Diseases (ICD) coding changes from ICD-9 to ICD-10 that took place before 1998. Admissions before 1998 were identified by using ICD-9 codes 995.0 (anaphylaxis, unspecified) and 995.6 (food-induced anaphylaxis). All hospital admissions from 1998 onward were included where the principal diagnosis corresponded to the following ICD-10 (international version) codes: anaphylactic shock due to adverse food reactions (T78.0); anaphylactic shock, unspecified (T78.2); and anaphylactic shock due to adverse effects of correct drug or medicament properly administered (T88.6). Hospitalizations in which a primary T78 code was associated with a secondary X23 code were classified as being caused by insect sting–related anaphylaxis. We also analyzed the data to assess the possible effect of the introduction of a maximum 4-hour wait in emergency departments by the United Kingdom (UK) Government in 2004.

### Fatal anaphylaxis data

All deaths in England and Wales are recorded by a medical doctor, and these data are collected by the Office for National Statistics (ONS). Since 1992, we (R.P. and M.H.G.) have collected data on all cases in which anaphylaxis was included as a cause of death. Cases were entered into a registry within the parameters permitted by the local research ethics committee and approved by the ONS. Additional notifications were collected from patient representative organizations, coroners, the police service, pathologists, and media reports, as previously described.^1^

### Verification of fatal anaphylaxis data

The attribution and coding of deaths can be unreliable, and therefore caution is needed when interpreting fatal anaphylaxis statistics. It is important that both the probability that a death was due to anaphylaxis and that the trigger for the reaction was correctly determined are taken into account when analyzing the data. Since the inception of the registry in 1992, we (R.P. and M.H.G.) have attempted to investigate the circumstances of every fatal anaphylactic episode using a previously outlined methodology.^1^ In brief, for each death, the probability that it was caused by anaphylaxis and the probability that the cause had been correctly identified were assessed. Deaths caused by an acute asthma exacerbation were included only where there was strong evidence that the episode was triggered by an identified allergen to which the deceased patient had a known allergy. Deaths caused by asphyxia from upper airways angioedema in patients with hereditary angioedema or in those taking angiotensin-converting enzyme inhibitors were excluded when an allergic cause for the reaction seemed improbable. We also excluded cases of amniotic fluid emboli (anaphylactoid syndrome of pregnancy). Because of difficulties in obtaining sufficient data to confirm the precise trigger for some cases of fatal anaphylaxis, particularly those caused by medication, we have included cases from the ONS database in which sufficient information was available to determine that anaphylaxis was the likely cause of death but not to confirm the specific cause (eg, medication) with high probability. We classified these as unconfirmed cases.

### Prescription of epinephrine autoinjector devices

We extracted data from the National Health Service Business Services Authority Prescription Cost Analysis databases from 1998 to 2012, which record all prescriptions issued by health practitioners through the English public health system. We grouped epinephrine autoinjector devices into 150- and 300-μg doses, irrespective of device. We were unable to obtain similar data for Wales.

### Statistics

Age-standardized rates (ASRs) for death and hospital admissions were calculated by standardizing to the age distribution of the population in mid-2001 (for 1992-2012) and mid-2006 (1998-2012), as reported by the ONS; thus cases (admissions or fatalities) are expressed per 100,000 population. Poisson regression was used to estimate the rate ratio for the annual increase in rates by calendar year, as previously described.^11^ Results are presented as rate ratios and 95% CIs. A rate ratio of 1.0 indicates no annual change in rate, and a 95% CI that includes 1.0 indicates the observed rate ratio is not statistically significant.

## Results

**Fig 1:**
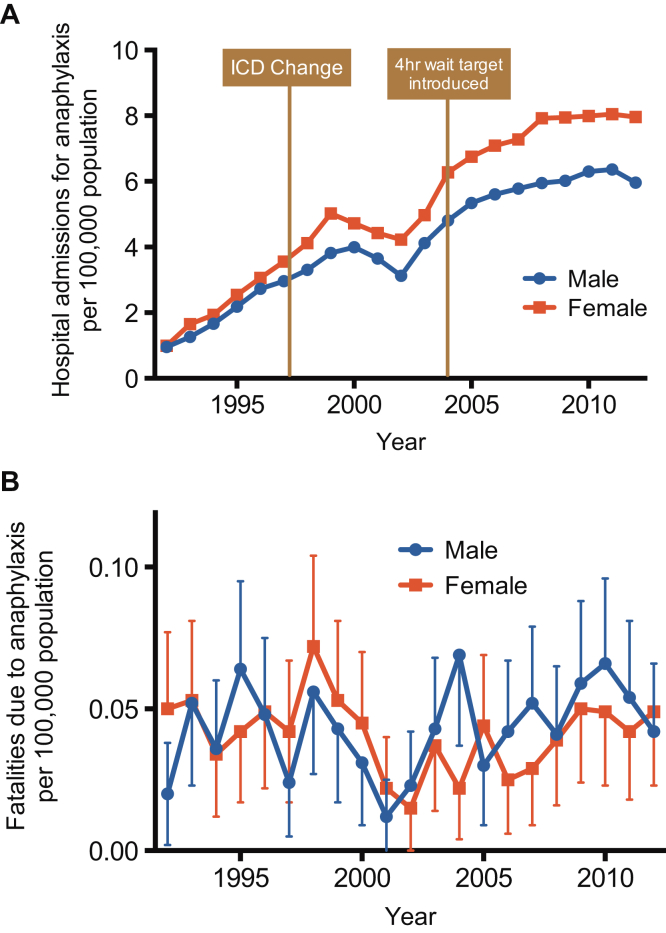
Time trends in hospital admissions **(A)** and fatalities **(B)** for all-cause anaphylaxis between 1992 and 2012. *Vertical bars* represent SEMs.

Hospital admissions because of all-cause anaphylaxis increased steadily from 1992 for both sexes but appear to have reached a plateau since 2008 (Fig 1, *A*). Over the study period, there was an increase in hospital admissions of 615%, from 1.0 to 7.0 cases per 100,000 population per annum. The estimated rate ratio (multiplicative increase of the rate per year over the study period) was 1.073 (95% CI, 1.071-1.075; *P* < .001). This trend was not clearly related to either a change in ICD coding (ICD-9 to ICD-10) or the introduction of a 4-hour maximum observation in emergency departments in the UK (Fig 1, *A*), which might have resulted in an increase in hospitalization. In contrast, fatality rates from all-cause anaphylaxis remained stable at a mean of 0.047 cases (95% CI, 0.042-0.052 cases) per 100,000 population per annum (Fig 1, *B*), with no increase in fatalities during this period (estimated rate ratio, 1.00; 95% CI, 0.98-1.01; *P* > .05). There was no difference in the distribution of fatal cases by sex (*P* > .05).

### Food-induced anaphylaxis: Admissions and fatalities

**Fig E1:**
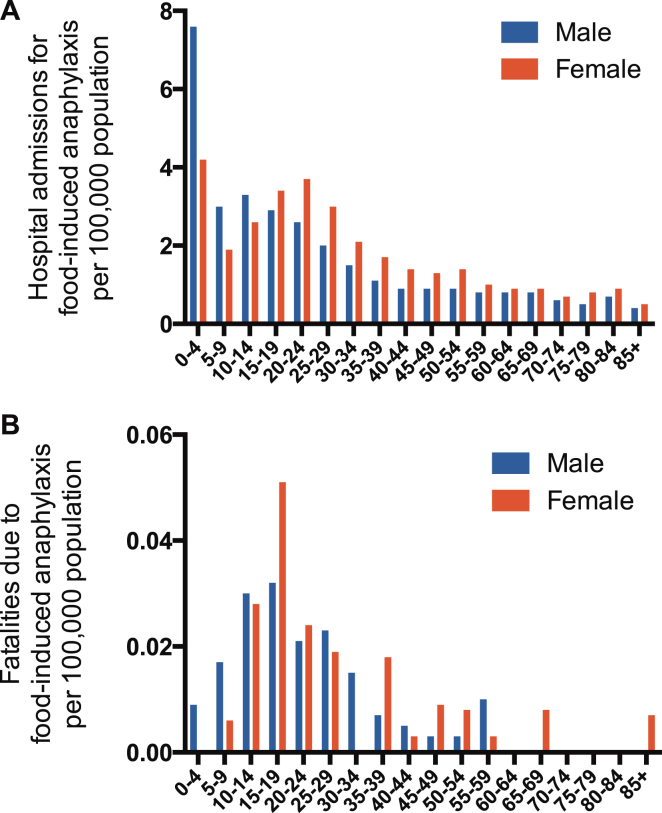
Distribution of hospital admissions **(A)** and fatalities **(B)** caused by food-induced anaphylaxis by age and sex.

**Fig 2:**
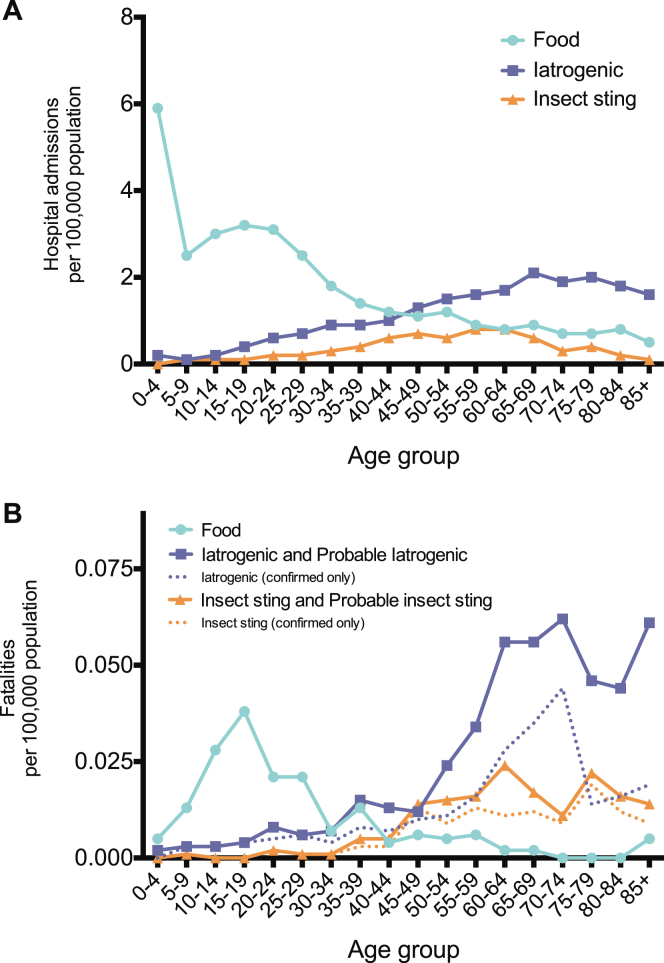
Age distribution of ASRs for admissions (1998-2012; **A**) and fatalities (1992-2012; **B**) caused by anaphylaxis by triggering agent (food, iatrogenic causes, and insect stings).

**Fig 3:**
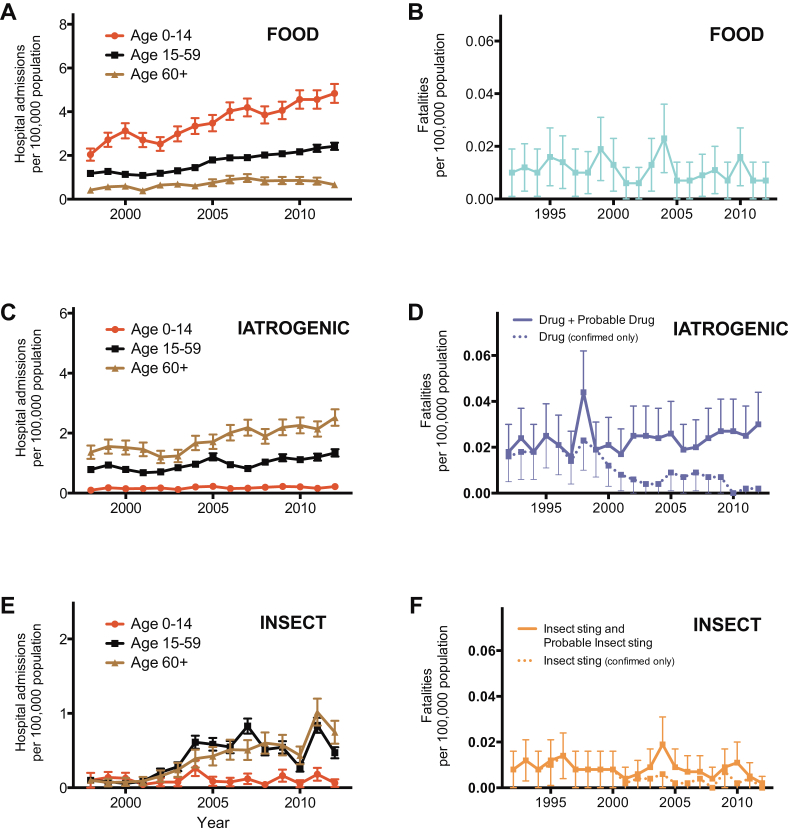
Time trend in ASRs for admissions to hospital with anaphylaxis **(A**, **C**, and **E)** and fatalities **(B**, **D**, and **F)** by trigger: food, iatrogenic causes, and insect stings. *Vertical bars* represent SEMs.

There were a total of 14,675 hospital admissions coded as anaphylaxis because of a food trigger between 1998 and 2012, with the highest rate in children and adults less than 24 years old (Fig 2, *A*). Hospital admissions increased significantly between 1998 and 2012, from an ASR of 1.2 to 2.4 per 100,000 population per annum, resulting in an increase of 106% (*P* < .0001; Fig 3, *A*). The estimated rate ratio was 1.05 (95% CI, 1.05-1.06) over the study period. The estimated increases in ASR for the age groups of 0 to 14 years, 15 to 59 years, and 60 or more years were 137% (rate ratio, 1.05; 95% CI, 1.05-1.06; *P* < .001), 105% (rate ratio, 1.06; 95% CI, 1.06-1.07; *P* < .001), and 58% (rate ratio, 1.04; 95% CI, 1.03-1.05; *P* < .001), respectively. There was no significant difference in the ASR by sex (*P* > .05; see Fig E1, *A*, in this article's Online Repository at www.jacionline.org). Male subjects outnumbered female subjects (1.7:1) among children less than 15 years of age, whereas female subjects outnumbered male subjects (1.4:1) among those older than 15 years (see Fig E1, *A*).

One hundred twenty-four fatalities were assessed as being highly likely to be caused by ingestion of a food allergen between 1992 and 2012. The mean age of fatal food-induced cases of anaphylaxis was 25 years (95% CI, 22-28 years). The ASR for fatal food-induced anaphylaxis was 0.011 cases (95% CI, 0.009-0.013 cases) per 100,000 population per annum, with a peak in the 10- to 29-year age groups (Fig 2, *B*). This rate remained stable over the study period (estimated rate ratio, 0.99; 95% CI, 0.96-1.02; *P* > .05; Fig 3, *B*). We did not observe a significant difference in the age distribution of fatal cases by sex (*P* > .05, see Fig E1).

**Fig 4:**
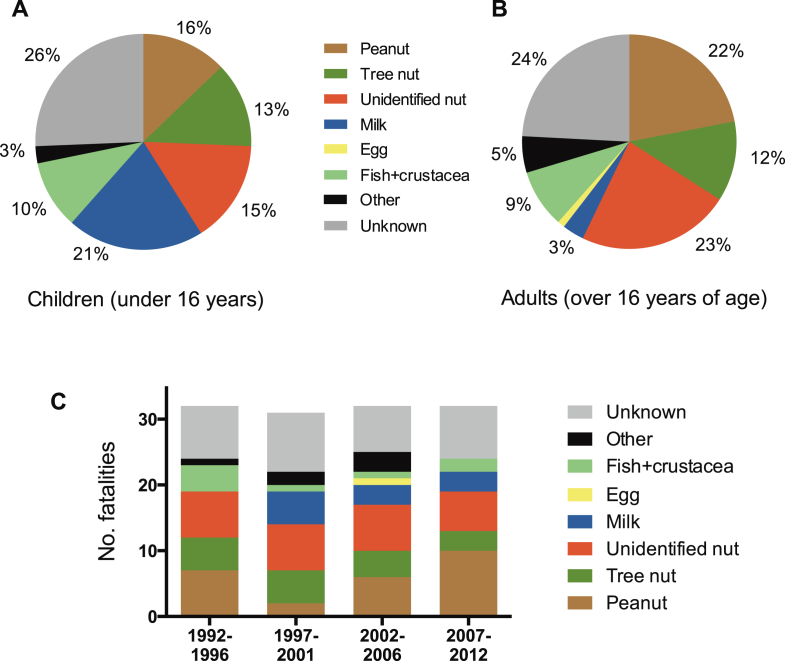
Cause of fatal food-induced anaphylaxis cases by trigger in children **(A)** and adults **(B)** and by 5-year groups **(C)**.

The triggering food was recorded as identified in 95 (77%) of 124 cases (Fig 4, *A* and *B*). Of these, 69 (73%) of 95 fatalities were caused by allergy to peanut or tree nuts. Cow's milk accounted for 8 (21%) of 39 fatalities in children less than 16 years of age (Fig 4, *A*). Ninety-seven (78%) of 124 fatal cases were in patients with a physician's diagnosis of asthma, 86 (69%) were known to have a food allergy before the fatal event, and at least 26 (21%) had experienced prior anaphylaxis. Thirty-three (27%) fatalities were triggered by allergen ingestion in the patient's own home, whereas 25 (20%) occurred in restaurants. Among school-age children (age, 4-18 years), 8 (17%) of 48 fatalities occurred within the educational environment. The source of the food was identified in 100 cases: 27 (27%) were caused by the consumption of the allergen in prepackaged foods. Fifty-nine (59%) reactions were to food products provided by a catering establishment, of which one quarter were purchased from takeaway outlets.

### Anaphylaxis of iatrogenic causes: Admissions and fatalities

A total of 8161 admissions to the hospital were coded as iatrogenic cause–related anaphylaxis between 1998 and 2012, with the highest admission rates seen from the seventh decade onward (Fig 2, *A*). Admissions to the hospital increased significantly from an ASR of 0.78 to 1.4 per 100,000 population per annum, an increase of 82% (Fig 3, *C*). The estimated rate ratio was 1.04 (95% CI, 1.04-1.05; *P* < .001) over the study period. The mean ASR in the 0- to 14-year age group was stable throughout the study period at 0.17 (95% CI, 0.16-0.20) admissions per 100,000 population per annum (no significant increase, *P* > .05). This is in contrast to the age groups of 15 to 59 years and 60 years and older: the estimated increase in ASR for those 15 to 59 years and those 60 or more years of age were 71% (rate ratio, 1.04; 95% CI, 1.03-1.04; *P* < .001) and 85% (rate ratio, 1.05; 95% CI, 1.04-1.05; *P* < .001), respectively.

Two hundred sixty-three fatalities were recorded as iatrogenic cause–related anaphylaxis between 1992 and 2012. The mean age of fatal cases of anaphylaxis of iatrogenic causes was 58 years (95% CI, 56-61 years), with fatalities rare before the fourth decade of life. The overall ASR of fatal drug-induced anaphylaxis was 0.024 (95% CI, 0.021-0.026) per 100,000 population per annum, peaking in the seventh decade of life onward (Fig 2, *B*). This rate remained stable over the study period (estimated rate ratio, 1.01; 95% CI, 0.99-1.03; *P* > .05; Fig 3, *D*).

### Insect sting–induced anaphylaxis: Admissions and fatalities

Between 1998 and 2012, there were 2688 admissions to the hospital because of anaphylaxis caused by insect stings (Fig 2, *A*). Admissions were rare in the first 3 decades of life. There was a significant increase in admissions during the study period, from an ASR of 0.1 to 0.5 per 100,000 population per annum (*P* < .001; Fig 3, *E*). The estimated rate ratio was 1.12 (95% CI, 1.10-1.13), which is equivalent to an increase of 410% over the study period. This increase was due to an increase in ASR in the 15- to 59-year and 60-year and older age groups, both of which saw an increase in admissions with an estimated rate ratio of 1.11 (95% CI, 1.09-1.12; *P* < .001) and 1.17 (95% CI, 1.14-1.19; *P* < .001), respectively. The ASR in the 0- to 14-year age group remained stable during this time (estimated rate ratio, 0.99; 95% CI, 0.95-1.04; *P* > .05).

We identified 93 deaths caused by sting-induced anaphylaxis between 1992 and 2012. The mean age of fatal cases was 59 years (95% CI, 56-63 years), with fatalities rare before the fifth decade of life. The mean ASR for fatal insect sting–induced anaphylaxis was 0.009 (95% CI, 0.007-0.010) per 100,000 population per annum (Fig 2, *B*). This rate remained stable over the study period (estimated rate ratio, 0.98; 95% CI, 0.94-1.01; *P* > .05; Fig 3, *F*).

### Differences in age distribution of fatal anaphylaxis by trigger

Fatalities were most common in older persons for drug- and insect sting–induced anaphylaxis, whereas for food-triggered reactions, fatalities peaked in the second and third decades (*P* < .01, Mann-Whitney test).

**Fig 5:**
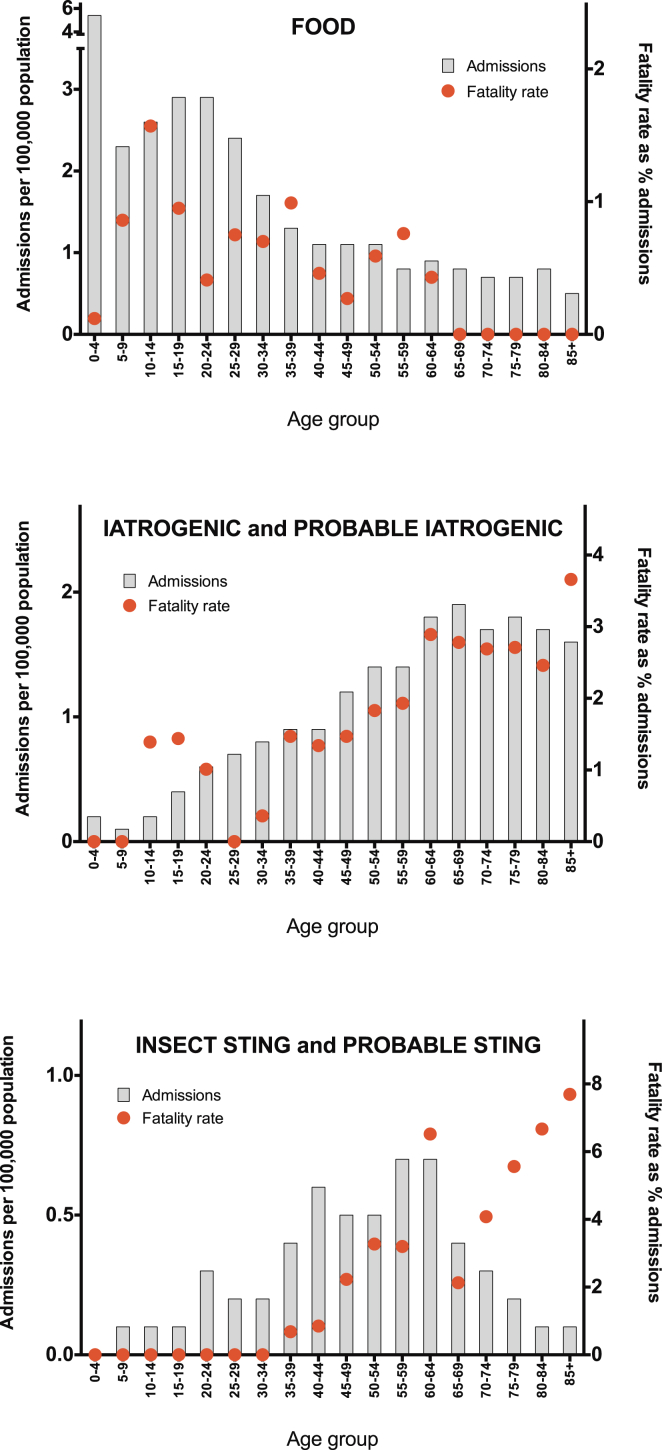
Incidence of fatal anaphylaxis expressed as a proportion of hospital admissions caused by anaphylaxis by trigger.

To assess whether the different age distribution of fatal food-induced anaphylaxis might be explained by age-related differences in the prevalence of food-induced anaphylaxis, we attempted to normalize the fatality data by expressing them as a proportion of all hospital admissions for that age group (as a surrogate measure for the incidence of severe allergic reactions), as shown in Fig 5. In contrast to fatal outcomes from drug- and insect sting–induced anaphylaxis, which increased with age, fatal outcomes from food-induced anaphylaxis were most common in the second decade of life and very rare in adults older than 60 years.

### Provision of epinephrine autoinjector devices

**Fig 6:**
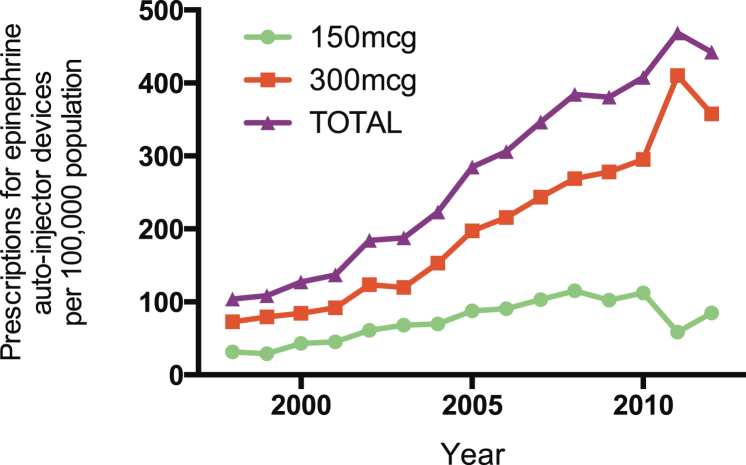
Prescription of epinephrine autoinjectors to English residents from 1998 to 2012.

The prescription of epinephrine autoinjector devices to residents in England increased by 325% from 1998 to 2012 (*P* < .0001, Fig 6). The estimated rate ratio was 1.113 (95% CI, 1.112-1.113) over the study period.

## Discussion

To our knowledge, this is the largest data set of fatal anaphylaxis cases reported, which allows more robust conclusions regarding trends in fatal anaphylaxis to be made compared with previous studies. Hospital admissions for anaphylaxis in England and Wales increased 7-fold from 1992 to 2012, although the incidence might have reached a plateau over the last 5 years. We found no evidence for an increase in fatal anaphylaxis over the same period, suggesting that changes in the recognition and management of anaphylaxis might explain some of the observed increase in anaphylaxis-related hospitalizations. These findings are similar to those reported for the United States from an analysis of health databases (which might be subject to inclusion bias) over a shorter period of time.^12^ However, we also analyzed trends in anaphylaxis admissions and fatalities by cause in a national population over 2 decades: we found that the age distribution of anaphylaxis resulting in hospital admission and fatal anaphylaxis varies significantly according to trigger.

Of note, the ASRs for hospital admissions caused by anaphylaxis in our study are consistent with those previously reported for Australia,^13^ New York State,^14^ and the United States.^12^ Although there has been an increase in all-cause hospital admissions over the past decade in England,^15^ the increase in anaphylaxis-related admissions is far greater than that reported for all-cause admissions. Therefore we consider that the increase observed is not accounted for by the general trend in increasing emergency admissions discussed elsewhere.^15^

Our finding that hospitalizations for anaphylaxis have increased steadily while fatality rates have remained stable has several possible explanations. Improved recognition/coding/awareness of anaphylaxis might have led to an increase in patient attendance/hospital admission/diagnostic coding without a real increase in disease incidence. The rate of anaphylaxis might have increased (mirroring the increase in prevalence of atopy reported elsewhere^16^), but the management of severe allergic reactions has improved at the same time, resulting in a reduced case fatality rate. In support of this, we show that prescriptions for epinephrine autoinjectors have increased over the same time period. However, arguing against a protective role for epinephrine autoinjectors is our previous observation that up to one third of fatal food-induced anaphylactic episodes in the UK occur despite prompt administration of intramuscular epinephrine^17^ and our current finding of increased admissions without any increase in fatalities for anaphylaxis due to iatrogenic causes (a situation in which epinephrine autoinjectors are not commonly prescribed). It is possible that other aspects of anaphylaxis management have improved sufficiently such that no increase in the case fatality rate was observed. A final possibility is that although anaphylaxis has increased in incidence, this increase is of a type of anaphylaxis that does not result in fatalities; however, we consider this to be unlikely.

Diagnosis of anaphylaxis might be more difficult in very young children and more prone to misclassification, and therefore it is possible that the peak in the rate of hospitalization for food-induced anaphylaxis in early childhood is partly artifactual (particularly in view of the low rate of fatalities in younger children). In 2011, new national guidelines were released that recommended 6 to 12 hours of observation after an anaphylactic episode and hospital admission for children less than 16 years of age (but not adults) with anaphylaxis^18^; however, the majority of the data presented here precede this advice. Food allergy is most prevalent in infants and young children, and preschool children with food allergy are known to have a high frequency of allergic reactions^19^; therefore it is likely that food-induced allergic reactions in general are most common in preschool children. The age- and sex-specific distribution of hospital admissions for food-induced anaphylaxis in England and Wales is very similar to what has been previously reported in Australia.^13^ The increasing trend for antibiotic prescriptions in the UK, with the highest rates of antibiotic prescribing occurring in older age groups,^20^ might explain (at least in part) both the increase in anaphylaxis of iatrogenic cause over time and the age distribution observed.

Fatalities and admissions were most common in those 60 years of age and older for iatrogenic cause– and insect sting–induced anaphylaxis, which is in contrast to food-induced anaphylaxis, for which fatalities were most common in the second and third decades. These observations might be confounded by age-related differences in the incidence of food-induced allergic reactions; that is, a lower incidence of fatal, food-triggered reactions in older adults might reflect a lower incidence of food-induced anaphylaxis in this age group. We attempted to account for this by normalizing the data as a proportion of hospital admissions. We found that the age-related differences in fatal anaphylaxis by causative trigger are not explained by differences in the rate of less severe allergic reactions, a finding we have also noted for anaphylaxis in Australia.^21^

It has been proposed that the increased risk of food-induced anaphylaxis-related fatalities in teens and younger adults might be due to risk-taking behaviors.^22,23^ However, such factors are less likely to be relevant to adults in the fourth decade of life, an age group we have shown is still associated with a high risk, although cofactors (eg, exercise or alcohol consumption) might affect this age group more than others. Therefore our data raise the possibility that food-induced anaphylaxis might not have the same physiologic basis as anaphylaxis triggered by insect stings or iatrogenic causes. It is noteworthy that symptoms of anaphylaxis vary according to trigger in fatal,^1^ severe (with hypoxemia and/or hypotension),^24^ and less severe^25^ reactions. Cardiovascular compromise is common in patients with severe reactions to drugs and insect venom^1,9,24,25^ but not those with food-triggered reactions, in whom life-threatening manifestations are generally caused by laryngopharyngeal and/or respiratory compromise.^1,9^ Where cardiovascular arrest occurs in food-triggered reactions, this is generally thought to be secondary to respiratory arrest.^1^ In our series more than 75% of patients with fatalities caused by food-triggered anaphylaxis had asthma. In a large prospective study of severe anaphylaxis in an unselected cohort presenting for emergency care in Australia, underlying lung disease was predictive of severe respiratory involvement.^24^ Furthermore, many patients with food allergy without a formal diagnosis of asthma have underlying bronchial hyperreactivity,^26^ which can predispose to more severe reactions.^27^ However, the age distribution of fatal asthma is different to that seen here for fatal food-induced anaphylaxis,^28^ suggesting that the vulnerability of teenagers and younger adults to severe food-induced anaphylaxis is not directly related to an age-related predisposition to fatal bronchospasm. Our data suggest that there might be other factors, potentially other age-related physiologic processes, that account for the vulnerability of this age group to fatal outcomes from food-induced allergic reactions. We did not find any significant sex-related differences in the age distribution for fatal food-induced anaphylaxis, suggesting that any influences are not sex specific. Investigating the nature of these physiologic influences through mechanistic assessments might lead to important advances in our understanding of determinants of severity in patients with food-induced allergic reactions.

We observed a clear increase in fatalities with age for anaphylaxis of iatrogenic causes and that caused by insect stings. This could be explained by a combination of age-related increased prevalence of allergy to these triggers, as well as increased susceptibility to anaphylaxis at older ages. Anaphylaxis triggered by iatrogenic causes and insect stings are more likely to result in cardiovascular involvement,^1,9,24,25^ perhaps because of more rapid exposure of allergen (for parenteral medication and venom) to the cardiovascular system. However, arguing against this is the observation that food allergens are rapidly absorbed across the oral mucosa, resulting in plasma levels sufficient to trigger an effector cell response,^29^ and thus might be expected to cause both cardiovascular and respiratory symptoms. Furthermore, severe allergic reactions to oral medication (eg, antibiotics) are frequently associated with cardiovascular manifestations,^24,25^ which is more in common with anaphylaxis caused by parenterally administered medication in terms of symptoms elicited. The increased mortality in older age groups could represent an age-related susceptibility to anaphylaxis-induced cardiovascular effects, including, but not limited to, a possible role for cardiac mast cells.^30^ Younger patients might be more able to compensate for a cardiovascular insult during an anaphylactic reaction, thus explaining the relative absence of cardiovascular symptoms in patients with fatal food-induced anaphylaxis. However, this cannot completely explain the differences in age-related susceptibility to fatal outcome between different triggers of anaphylaxis seen in our study.

The strength of this study is that we used a large population-based data set, which allows for more detailed analysis of time trends and age/sex distribution of anaphylaxis by cause. However, our data do have some limitations. The cause of hospitalization for anaphylaxis might be miscoded, with either the eliciting trigger or even the diagnosis being incorrect. To limit this, we used diagnoses assigned at discharge by the medical team after the patient would have been reviewed by at least one senior physician. Furthermore, we used a similar methodology throughout the study period, and therefore our data are useful in terms of monitoring time trends in hospital admissions caused by anaphylaxis. We did not include cases of anaphylaxis seen in emergency departments that did not require hospitalization because these data sets are incomplete and very prone to miscoding.^31^ Similarly, we could not include a population-based assessment of the prevalence of anaphylaxis in consumers who do not present to emergency services, something that is common.^23^ With regard to fatalities, cases were registered on an independent and dedicated registry and only included where the balance of probabilities suggested anaphylaxis was the likely cause. Because of a concern that some food-triggered deaths might have been wrongly attributed to asthma in causality during hospital certification,^32^ we (R.P. and M.H.G.) undertook a separate study during 2003 and 2004, when all asthma deaths in the UK up to the age of 32 years were studied prospectively for a 12-month period to determine whether the fatal attack had been triggered by food allergy. On the basis of this research and others, it is unlikely that cases of fatal food-induced anaphylaxis were miscoded as fatal asthma in this data set.^17,28^

In summary, our data show that the rate of hospitalization for anaphylaxis has increased markedly in England and Wales over a 21-year period, yet the rate of fatal anaphylaxis remains stable. We have also identified marked differences in the age distribution of anaphylaxis admissions and fatalities according to trigger, which might offer important clues to the underlying pathophysiology. Further investigation of age-related vulnerability to anaphylaxis can enhance our understanding of factors that predispose to poor outcomes in patients with severe allergic reactions.

**Key messages**

• Hospitalizations for anaphylaxis increased between 1992 and 2012, but rates of fatal anaphylaxis did not.
• The age distributions of anaphylaxis resulting in hospital admission and fatal anaphylaxis vary significantly according to trigger.
• Fatal food-induced anaphylaxis is most common during the second and third decades of life.

## References

[1] Pumphrey R.S. (2000). Lessons for management of anaphylaxis from a study of fatal reactions. Clin Exp Allergy.

[2] Cummings A.J., Knibb R.C., King R.M., Lucas J.S. (2010). The psychosocial impact of food allergy and food hypersensitivity in children, adolescents and their families: a review. Allergy.

[3] Lau G.Y., Patel N., Umasunthar T., Gore C., Warner J.O., Hanna H. (2014). Anxiety and stress in mothers of food-allergic children. Pediatr Allergy Immunol.

[4] Poulos L.M., Waters A.M., Correll P.K., Loblay R.H., Marks G.B. (2007). Trends in hospitalizations for anaphylaxis, angioedema, and urticaria in Australia, 1993-1994 to 2004-2005. J Allergy Clin Immunol.

[5] Wood R.A., Camargo C.A., Lieberman P., Sampson H.A., Schwartz L.B., Zitt M. (2014). Anaphylaxis in America: the prevalence and characteristics of anaphylaxis in the United States. J Allergy Clin Immunol.

[6] Turner P.J., Boyle R.J. (2014). Food allergy in children: what is new?. Curr Opin Clin Nutr Metab Care.

[7] Umasunthar T., Leonardi-Bee J., Hodes M., Turner P.J., Gore C., Habibi P. (2013). Incidence of fatal food anaphylaxis in people with food allergy: a systematic review and meta-analysis. Clin Exp Allergy.

[8] Gibbison B., Sheikh A., McShane P., Haddow C., Soar J. (2012). Anaphylaxis admissions to UK critical care units between 2005 and 2009. Anaesthesia.

[9] Worm M., Moneret-Vautrin A., Scherer K., Lang R., Fernandez-Rivas M., Cardona V. (2014). First European data from the network of severe allergic reactions (NORA). Allergy.

[10] Bock S.A., Muñoz-Furlong A., Sampson H.A. (2001). Fatalities due to anaphylactic reactions to foods. J Allergy Clin Immunol.

[11] Frome E.L., Checkoway H. (1985). Epidemiologic programs for computers and calculators. Use of Poisson regression models in estimating incidence rates and ratios. Am J Epidemiol.

[12] Ma L., Danoff T.M., Borish L. (2014). Case fatality and population mortality associated with anaphylaxis in the United States. J Allergy Clin Immunol.

[13] Liew W.K., Williamson E., Tang M.L. (2009). Anaphylaxis fatalities and admissions in Australia. J Allergy Clin Immunol.

[14] Lin R.Y., Anderson A.S., Shah S.N., Nurruzzaman F. (2008). Increasing anaphylaxis hospitalizations in the first 2 decades of life: New York State, 1990-2006. Ann Allergy Asthma Immunol.

[15] Blunt I., Bardsley M., Dixon J. (2010). Trends in emergency admissions in England 2004–2009. http://www.nuffieldtrust.org.uk/sites/files/nuffield/Trends_in_emergency_admissions_REPORT.pdf.

[16] Prescott S.L., Pawankar R., Allen K.J., Campbell D.E., Sinn J.K., Fiocchi A. (2013). A global survey of changing patterns of food allergy burden in children. World Allergy Organ J.

[17] Pumphrey R.S., Gowland M.H. (2007). Further fatal allergic reactions to food in the United Kingdom, 1999-2006. J Allergy Clin Immunol.

[18] (2011). Anaphylaxis: assessment to confirm an anaphylactic episode and the decision to refer after emergency treatment for a suspected anaphylactic episode.

[19] Fleischer D.M., Perry T.T., Atkins D., Wood R.A., Burks A.W., Jones S.M. (2012). Allergic reactions to foods in preschool-aged children in a prospective observational food allergy study. Pediatrics.

[20] Wrigley T., Majeed A. (2002). Age- and sex-specific antibiotic prescribing patterns in General Practice in England and Wales, 1994 to 1998. http://www.ons.gov.uk/ons/rel/hsq/health-statistics-quarterly/no%1314%13summer-2002/age-and-sex-specific-antibiotic-prescribing-patterns-in-general-practice-in-england-and-wales%131994-to-1998.pdf.

[21] Turner P.J., Sharma V., Tang M.L.K., Gowland M.H., Harper N., Garcez T. (2014). Age As a Risk Factor For Fatal Food-Induced Anaphylaxis: An Analysis Of UK and Australian Fatal Food Anaphylaxis Data [abstract]. J Allergy Clin Immunol.

[22] Sampson M.A., Muñoz-Furlong A., Sicherer S.H. (2006). Risk-taking and coping strategies of adolescents and young adults with food allergy. J Allergy Clin Immunol.

[23] Noimark L., Wales J., Du Toit G., Pastacaldi C., Haddad D., Gardner J. (2012). The use of adrenaline autoinjectors by children and teenagers. Clin Exp Allergy.

[24] Brown S.G., Stone S.F., Fatovich D.M., Burrows S.A., Holdgate A., Celenza A. (2013). Anaphylaxis: clinical patterns, mediator release, and severity. J Allergy Clin Immunol.

[25] Worm M., Edenharter G., Ruëff F., Scherer K., Pföhler C., Mahler V. (2012). Symptom profile and risk factors of anaphylaxis in Central Europe. Allergy.

[26] Kivity S., Fireman E., Sade K. (2005). Bronchial hyperactivity, sputum analysis and skin prick test to inhalant allergens in patients with symptomatic food hypersensitivity. Isr Med Assoc J.

[27] Krogulska A., Dynowski J., Wasowska-Królikowska K. (2010). Bronchial reactivity in schoolchildren allergic to food. Ann Allergy Asthma Immunol.

[28] (2014). National Review of Asthma Deaths.

[29] Dirks C.G., Pedersen M.H., Platzer M.H., Bindslev-Jensen C., Skov P.S., Poulsen L.K. (2005). Does absorption across the buccal mucosa explain early onset of food-induced allergic systemic reactions?. J Allergy Clin Immunol.

[30] Genovese A., Rossi F.W., Spadaro G., Galdiero M.R., Marone G. (2010). Human cardiac mast cells in anaphylaxis. Chem Immunol Allergy.

[31] (2008). Accident and Emergency Attendances in England (Experimental Statistics) 2007-08.

[32] Pumphrey R.S., Roberts I.S. (2000). Postmortem findings after fatal anaphylactic reactions. J Clin Pathol.

